# Optimizing the configuration of deep learning models for music genre classification

**DOI:** 10.1016/j.heliyon.2024.e24892

**Published:** 2024-01-17

**Authors:** Teng Li

**Affiliations:** Academy of Arts, Pingdingshan Polytenchnic College, Pingdingshan, 467000, Henan, China

**Keywords:** Deep reinforcement learning, Convolutional neural network, Signal processing, Music genre classification

## Abstract

Music genre categorization is a fundamental use of sound processing methods in the realm of music retrieval. Typically, people are responsible for categorizing music genres. Machine learning approaches can automate this procedure. Therefore, in recent years, several approaches have been suggested to achieve this objective. Nevertheless, the given findings indicate that there is still a discrepancy between the observed results and an optimal categorization method. Hence, this paper introduces a novel approach for accurately forecasting music genres by using deep learning methodologies. The proposed approach involves preprocessing the input signals and then representing the characteristics of each signal using a combination of Mel Frequency Cepstral Coefficients (MFCC) and Short-Time Fourier Transform (STFT) features. Subsequently, a convolutional neural network (CNN) is applied to process each group of these characteristics. The proposed technique utilizes two CNN models to analyze MFCC and STFT data. Although the structure of these models is identical, the hyper-parameters of each model are individually adjusted using the black hole optimization (BHO) algorithm. Here, the optimization method fine-tunes the hyperparameters of each CNN model to minimize their training error. Ultimately, the results of two Convolutional Neural Network (CNN) models are combined to determine the music genre using a classifier based on SoftMax. The efficacy of the suggested methodology in categorizing music genres has been assessed using the GTZAN and Extended-Ballroom datasets. The experimental findings demonstrated that the suggested approach achieved classification accuracies of 95.2 % and 95.7 % in the two datasets, respectively, indicating its superiority over earlier efforts.

## 1 Introduction

Genre can be considered as one of the main criteria for classifying music. Identifying the genre of a music should be done based on attributes such as: instruments, musical techniques, cultural background and its content features [1]. In some cases, geographical characteristics are also used to distinguish the genre of music; although a genre based on geographical location often includes several sub-genres [2]. In general, today, music genres are recognized by humans. This process is very time-consuming and exhausting in large music databases and can be affected by the error of human factors [3]. Given the proliferation of online storage and large-scale databases, there is an increasing need for automated methods to accurately classify music genres. In recent years, machine learning approaches have garnered significant interest from academics and have been used to address a multitude of challenges. Recognizing musical genres is one of the issues that is well adapted to classification and machine learning techniques [4]. In recent years, several methods have been presented for automatic recognition of music styles. Initially, the recognition process was based on basic learning models such as decision tree [5], support vector machine (SVM) [6] and artificial neural networks (ANNs) [7]. Subsequently, when deep learning techniques were more widely used and models based on these techniques proved very effective in solving many problems, the number of approaches proposed for identifying musical genres using deep learning models grew, leading to greater popularity. The domain of machine learning is still expanding [8]. The analysis of these studies demonstrates that although deep learning approaches have really improved classification accuracy in comparison to prior methods, they still fall short of an optimal genre categorization system. Conversely, a fundamental challenge with deep learning models is their intricate structure, which poses difficulties in determining the optimal configuration for these models [9]. For this reason, most of the previous researches about music genre classification based on deep learning cannot guarantee the highest achievable performance. Attempting to provide a model with optimal self-configuration capabilities for music genre classification applications has motivated the current research. In this research, an attempt has been made to solve this problem by determining the optimal (or close to optimal) configuration for deep learning models. The proposed method, models this goal as an optimization problem and tries to minimize the training error of these learning models by adjusting the hyperparameters of CNN models. The contribution of this paper is twofold: In this article, a hybrid and parallel method for describing the characteristics of the signal is presented. In this strategy, the combination of MFCC and STFT features is used to comprehensively extract signal features. In this case, each of the MFCC and STFT features is processed by an independent CNN model to extract the signal features in a more efficient way. At the end, the set of features obtained from these CNN models are concatenated so that based on them, the characteristics of the signal can be described in a more comprehensive way. Also, in this article a new method for classifying music genres is presented, which uses the combination of convolutional neural networks and black hole optimization algorithm. In this method, hyperparameters of CNN models are adjusted based on the input features and using the black hole algorithm. The target hyperparameters for optimization in CNN models include: filter characteristics and the type of activation function in the intermediate layers. The use of this solution makes it possible to achieve a learning model with the lowest training error. This research aims to fulfill three objectives. The first objective is to study and evaluate the recent deep learning algorithms for classification of music genre using existing music databases. The second study aim involves assessing the effectiveness of the proposed deep learning algorithms in classifying music genres using pre-existing music datasets. The final purpose of this study is to compare the performance of the suggested deep learning algorithms with contemporary algorithms, after completing both of the previous objectives. The subsequent sections of this article are structured in the following manner: The second portion is dedicated to examining past efforts in the domain of music genre categorization. Subsequently, the third part elucidates the intricacies of the suggested methodology, followed by a comprehensive analysis of the outcomes derived from its application. Ultimately, the findings of the investigation are succinctly outlined in the fifth part.

## 2 Literature review

The fundamental procedure of automated techniques for categorizing music genres include the following stages: pre-processing, feature extraction, and classification. Meanwhile, several approaches have used feature selection techniques to decrease the size of signal characteristics. In recent years, many researches have attempted to solve this problem, and some of these methods are reviewed in this section. In Ref. [10], a method for classifying music genres using deep learning techniques is presented. In this method, first the features of the signal are extracted as a spectrogram matrix. Then the spectrogram matrix is reshaped and used as an input of a CNN model. This CNN model includes an input layer, 6 convolution layers, a fully connected layer and a classification layer.

A novel model is introduced in Ref. [11] to enhance the performance of Convolutional Neural Networks (CNNs) in music genre categorization. The Bottom-up Broadcast Neural Network (BBNN) model incorporates the long-term context aspects of the signal by propagating the output of each layer to all subsequent levels. In this method, the spectrogram matrix is used as the input of the BBNN model. Using this solution causes a significant increase in training time, but it increases the classification accuracy compared to the basic CNN models. In Ref. [12], a deep learning strategy based on the attention mechanism has been used to classify music genres. This method has presented a classification model based on the attention mechanism and Bi-directional Recurrent Neural Network (BiRNN) in order to consider the time differences of the spectrum in different time steps. Also, two serial and parallel models have been proposed based on the attention mechanism, and based on the results, the parallel model reports a higher performance.

Deep learning algorithms were used in Ref. [13] to categorize music genres and suggest music based on similarities of genres. The spectrogram picture serves as the input for a Convolutional Neural Network (CNN) model in this approach. This CNN architecture comprises of three convolutional layers and may serve the purposes of both classification and feature extraction. The findings indicate that the optimal performance of this strategy is attained using the CNN model for feature extraction and then feeding the retrieved features into an SVM model for classification. The study conducted in Ref. [14] used deep learning methods to examine the efficacy of several variables in enhancing the precision of music genre identification. This research demonstrates that using Mel scale features and Swaragram features may significantly enhance the classification accuracy of deep learning models. The research done in Ref. [15] uses the Parallel Recurrent CNN (PRCNN) model to recognize music genres. The PRNN model includes a CNN network and a BiRNN network that process the STFT matrix of the input signal in parallel and extract its features as a 256-length vector. This model utilizes Convolutional Neural Network (CNN) to extract spatial data, while Bidirectional Recurrent Neural Network (BiRNN) captures the temporal aspects of the input. Subsequently, the two sets of characteristics are merged to execute music genre identification using a SoftMax layer. In Ref. [16], a web-based music genre recognition system is presented that uses machine learning techniques. In this method, the MFCC matrix is used to describe the features of the signal. These features have been used as inputs for learning models, which include: Naive Bayes, Feedforward Neural Network, RNN and SVM.

The paper introduces a technique, described in Ref. [17], for categorizing music genres specifically designed for use in Musical Instrument Digital Interface (MIDI) files. This approach involves an initial pre-processing of the MIDI files, followed by the use of a feature extraction technique known as Pitch2Vec to represent the musical characteristics in a vectorized manner. Finally, a Masked Predictive Encode (MPE) model based on bidirectional transformation is used to transform features, which is an unsupervised model for representing musical features. Compared to other models such as RNN, the MPE method causes parallelization in the time steps based on which the training time is reduced. In Ref. [18], a combination of audio image-based features, spectral features, and traditional audio features have been used to distinguish music genres. In this model, the auditory image is created based on the modeling of the human auditory system. Additionally, the logarithmic frequency spectrogram is used to depict the spectrum characteristics. This choice is made because it is better suited for presenting the attributes of the low frequency portion of the signal, as opposed to the linear frequency spectrogram. In Ref. [19], a study introduces five novel hybrid techniques for categorizing music genres. The classification methods used in this study are: the weighted visibility graph based elastic net sparse classifier (WVG-ELNSC), the sequential machine learning with stacked autoencoder (SDA), the Riemannian Alliance based tangent space-based mapping (RA-STM) with transfer learning technique, the Transfer SVM (TSVM), and a classifier combining Bidirectional Long Short Term Memory (BiLSTM) with Graphical Convolution Network (GCN), referred to as BAG. BAG demonstrates higher accuracy in classifying music genres compared to the other four classifiers. The paper introduces a technique for categorizing music genres using a Convolutional Neural Network (CNN) and the Self Adaptive Sea Lion Optimization (SA-SLnO) algorithm [20]. This approach involves first extracting the non-negative matrix factorization (NMF) features, pitch features, and STFT features from the signal. These characteristics are then combined and inputted into a CNN model. This CNN architecture comprises a series of two-dimensional convolutional layers with 16 filters and dimensions of 3 × 3. Subsequently, three fully connected layers with dimensions of 384, 384, and 10 are sequentially positioned. The SA-SLnO algorithm is used in this approach to optimize the weights of the CNN model. It is important to acknowledge that in this particular scenario, the quantity of optimization parameters will be somewhat extensive, resulting in a wide-ranging search space inside the optimization problem. Hence, there is uncertainty over whether the optimization algorithm's weight settings can effectively decrease the training error of the CNN model. Utilizing a mix of deep learning models to leverage their strengths may result in the development of very potent hybrid classifiers. These hybrid models may be customized to suit different categorization issues. Researchers in Ref. [21] proposed a hybrid model that combines Convolutional Neural Networks (CNN) and Long Short-Term Memory (LSTM) to accurately identify emotions in electroencephalogram (EEG) data. This combination exhibits superior performance in contrast to the scenario when either CNN or LSTM model is used alone for emotion identification.

## 3 Proposed model

**Fig. 1:**
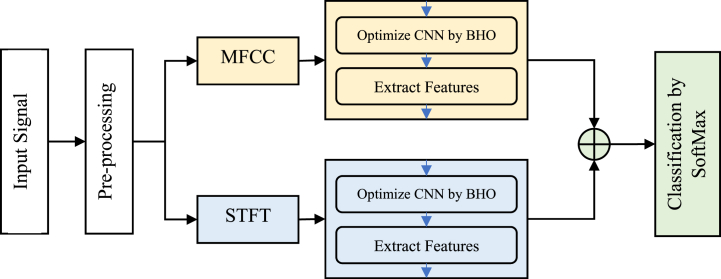
Block diagram of the proposed method.

The proposed method classifies music genres using two parallel CNN models, the hyperparameters of each of which are adjusted by the BHO algorithm. Inthis strategy, an attempt has been made to obtain a comprehensive model for describing musical stylistic features by simultaneously using MFCC and STFT features. The proposed method can be divided into the following main steps (Fig. 1):

i. Pre-processing of the initial signal
ii. Feature description based on MFCC and STFT
iii. Optimizing CNN models and extracting features
iv. Classification based on concatenated features

Fig. 1 illustrates that the first step involves pre-processing the input signals to ensure that all input samples are transformed into a standardized format with a consistent overall structure. This procedure involves the conversion of the signal's frequency and its subsequent normalization. The second phase involves extracting MFCC and STFT feature matrices from the pre-processed data. Each of these matrices is individually used as an input for a CNN model, where the values produced from the last completely connected layer are regarded as the ultimate extracted features. Prior to that, the BHO technique is used to finely tune the hyperparameters of each CNN model. The parameters of convolution filters, such as width, height, and number of filters, as well as the activation function used in each CNN model, are chosen to minimize the training error. In the subsequent process, the characteristics derived from CNN models are combined via a concatenation layer. This allows for the last phase of the proposed approach, where music genres are classified using a SoftMax-based classifier. The following section provides a detailed explanation of each individual phase of the suggested strategy.

### 3.1 Pre-processing

The suggested approach for categorizing music genres begins with the pre-processing of input data. The objective of this task is to mitigate the detrimental impact of certain specified circumstances in each sample, which might potentially result in misclassification. The frequency characteristics of the samples are one of the criteria included. Varying the frequency of an audio signal results in changes to the resolution of each signal's samples. In other words, increasing the frequency of a signal increases the number of samples per time unit. Since this feature cannot reflect anything about music genres; therefore, in the pre-processing step, first the frequency of all input signals is changed to a fixed value such as Fs. After doing this, two-channel (or more) signals are converted into mono-channel signals. This action causes each signal to be described in the form of a vector. At the end of the pre-processing step, all signals are converted into vectors with zero mean and unit variance. The signals obtained from the implementation of this step are used as the input of the second step of the proposed method.

### 3.2 Feature description

The second phase of the suggested approach involves describing the characteristics of the pre-processed signal using MFCC and STFT algorithms. This allows for the distinct use of each of these features in music genre categorization. Each of the listed strategies represents the characteristics of the incoming signal as a matrix.

#### 3.2.1 Feature description based on MFCC

The MFCC technique is modeled on the behavior of the human auditory system to analyze an audio signal. One of the reasons for the high efficiency of this technique is its high resolution. This means that minor changes in a signal can be well recognized through MFCC. Another strength of this method is the use of discrete cosine transform (DCT), which, while removing the details of the spectral structure, causes efficient summarization of the features. The calculation process for extracting the MFCC matrix from a signal includes the following steps [22]:

*Pre-emphasis filter:* The high-pass filter is applied to the input signal to remove unwanted spectral effects such as sudden changes in the input signal (which are caused by momentary intense noises) and make the signal uniform.

*Framing, windowing and overlapping:* During this stage, the signal is partitioned into smaller segments known as frames, and the characteristics of each frame are isolated. Typically, the duration of each frame falls within the range of 10–50 ms, and the frames are overlapping. The degree of overlap between the variable frames, namely within the range of 25 %–75 % of the frame length, is calculated. The frames obtained are subjected to multiplication inside a window to mitigate the impact of signal discontinuity at the start and end of each frame, as well as to avoid interference between frames in the frequency domain.

*Calculation of spectrum and filter bank in Mel scale:* In order to have easier and faster calculations, Fourier transform is used to transfer the speech signal to the frequency domain. In this step, the spectrum is estimated using the fast Fourier transform. In order to model the human auditory system in understanding sound frequencies, a non-linear transformation called Mel scale is used on the speech spectrum. This nonlinear transformation can model the sensitivity of the human auditory system to different frequency domains. In other words, the Mel scale shows that the human auditory system gives more importance to the information related to the lower domain, and for this reason, the signal spectrum is passed through 40 filters with the bandwidth of the Mel scale. These filters simulate the frequency resolution of the human auditory system. The filters are arranged in a triangle form, with each filter starting at the center frequency of the previous filter and ending at the center frequency of the next filter. Furthermore, the peak value of each filter is situated precisely at its central frequency. *Applying logarithmic and discrete* cosine *transform:* To reduce the number of components in the feature vector, the logarithmic values obtained from the above 40 filters are multiplied by DCT. The obtained result will be equal to the number of target MFCC coefficients. The output of this transformation is called Cepstral coefficients. This set of coefficients is more uncorrelated and the fewer components in it indicate the higher importance of information. On the other hand, more components in this vector have less information and are therefore less important.

*Calculation of derivatives of Cepstral coefficients:* Cepstral coefficients describe the set of signal characteristics well and can be effective in increasing the accuracy of recognition. To increase the accuracy of the detection system, we can derive these coefficients with respect to time. Actually, Cepstral coefficients model the static information in the signal and are sensitive to the changes in the signal. On the other hand, derivatives of Cepstral coefficients have dynamic information of transfer between different states. In this way, the combination of Cepstral coefficients and its derivatives together can be effective in increasing the richness of the features describing the signal [23].

#### 3.2.2 Feature description based on STFT

The second category of features used to describe the characteristics of the input signal is STFT. To do this, we consider the input signal as *N* consecutive frames such as f(m,n), where *m* indicates the frame ID and *n* indicates the sample ID in the current frame. Then, a STFT transformation is applied to each frame to obtain a vector with length *D* based on it. This transformation for each frame *f* in the signal can be formulated as follows [24]:

(1) $$F\left(m,k\right)=\sum_{n}f\left(m,n\right)e^{-\frac{j2\pi nk}{N}}$$

in the above relation, f(m,n) is calculated by equation (2):

(2) f(m,n)=f(n)ω(n−mS)

where, ω(n) is the windowing function for *N* samples and is located at the point mS; and in this case, *S* represents the step size in the samples. Also, *N* describes the number of discrete frequencies, which is determined using the fast Fourier transform (FFT) as a power-of-2. In this configuration, the overlapping rate between two consecutive frames is equal to $\frac{N-S}{N}$. Having F(m,k) by equation (1), the power spectral density (PSD) can be calculated by equation (3) [25]:

(3) $$P_{f}\left(m,k\right)=\frac{1}{N}{\left|F\left(m,k\right)\right|}^{2}$$

Considering the constant frequency of *Fs*, each frame is described in the form of *N* points that cover the frequency interval $\;[-\frac{Fs}{2},\frac{Fs}{2})$. Since the power spectrum is symmetrical, $\frac{N}{2}$ discrete frequency can be used to describe it.

### 3.3 Optimizing CNN models and extracting features

**Fig. 2:**
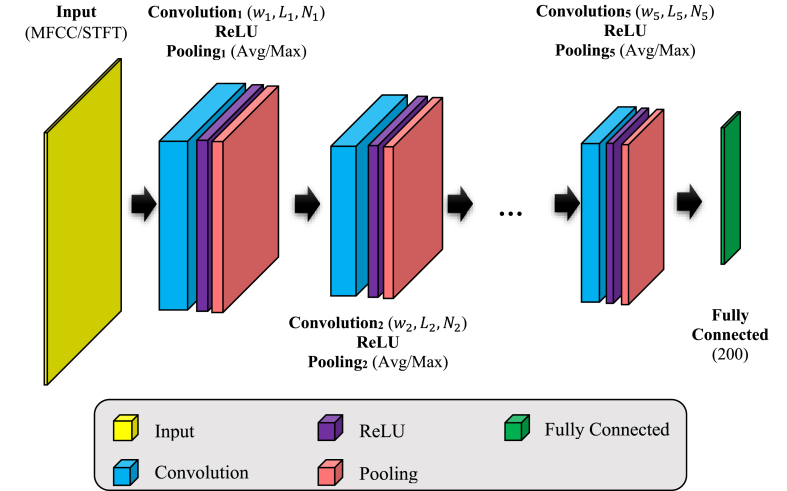
Structure of proposed CNN models for extracting MFCC and STFT features.

Following the description of the characteristics of each signal represented as MFCC and STFT matrices, two distinct CNN models are used to analyze each feature matrix. The overall architecture of these CNN models for handling both the MFCC and STFT features is comparable, with similar layer types and quantities. However, they vary in terms of the arrangement of hyperparameters. Since determining the optimal configuration for a CNN model is a time-consuming process and requires special accuracy, in the proposed method, the BHO algorithm is used to determine the optimal values for the hyperparameters of each CNN model. BHO is a nature-inspired optimization algorithm that aims to solve problems by mimicking the motion pattern of stars and black holes. It is a population-based algorithm known for its simplicity and efficient exploration of the problem space, making it a valuable tool for optimizing hyperparameters of the CCNs in current research. The feature of stars being swallowed by black holes in this algorithm can prevent solutions from being trapped in local optima. On the other hand, the simple mechanism of this algorithm and its simple operators lead to low memory consumption and high processing speed, which is of great importance in the problem discussed in this research. The general structure of proposed CNN models for processing MFCC and STFT matrices is shown in Fig. 2.

According to Fig. 2, each CNN model used in the proposed method includes an input layer, a fully connected layer and 5 convolution blocks. Each convolution block starts with a two-dimensional convolution layer and continues with ReLU and Pooling layers. The ReLU activation function in each convolution block passes only positive values and replaces negative values with zero. In this way, this function will generate output max (x,0) after receiving input x. Therefore, the operations performed in each convolution block can be formulated as equation (4):

(4) y=Pool(max(conv(I),0))

where, I and y determine the input and output of the convolution block, respectively. Also, conv (.) describes the convolution operation and Pool (.) shows the pooing operation of the block which can be defined as a max pooling or average pooling function. The input layer in each CNN model accepts the MFCC or STFT matrix obtained from the input signals. The fully connected layer at the end of the structure of each CNN model also contains the features extracted from the input matrices, which are described in the form of a vector with a length of 200. The depth of CNN models in this research was determined experimentally. Based on the experiments, using a depth greater than 5 for CNN models increases the risk of overfitting for some configurations (due to the relatively limited number of samples in the available databases). Meanwhile, a depth of less than 5 leads to a noticeable decrease in the accuracy of CNN models, which indicates model's under-fitting.

Both CNN models have identical static structures for their input and completely linked layers. However, the arrangement of the convolution blocks in the suggested approach is established using the BHO algorithm. The optimization process is used to establish the settings, such as the length, width, and number of filters in each convolution layer, as well as the kind of pooling function, in each convolution block. Therefore, in the suggested method, the most efficient arrangement of each CNN model is identified individually via the use of the BHO algorithm. Inthis procedure, the database is used to train samples, and each hyper-parameter of the structure is configured to minimize the training error. Once the suitable setup for each CNN model has been established, these models are used to analyze the test samples. The following part provides an explanation of the procedures involved in using the suggested BHO method to determine the ideal configuration for each CNN model. This includes a description of the optimization variables, solution vector structure, and fitness function. As mentioned, the purpose of using the BHO algorithm is to determine the optimal values for the hyperparameters of the convolution blocks in the CNN model. Each convolution block in the proposed CNN models includes the following configurable parameters:

• *Parameters of length, width and number of filters in each convolution layer*: In the design of the proposed CNN model, it is assumed that the length and width of the filter are equal in each convolution layer. Thus, each convolution block will have two adjustable parameters of size (length/width) and number of filters. In this case, the filter size parameter is described as a natural numerical variable in the interval [4,16]. Also, the filter count parameter can accept a natural number in the interval [4,64].
• *Pooling function type*: each pooling layer in the design of convolution blocks can choose one of the MaxPool or AveragePool functions. In the formulation of the optimization problem, for the Pooling function type parameter, the values 1 and 2 can be selected, in this case, the number 1 indicates the use of the MaxPool function and the number 2 indicates the allocation of the AveragePool function.

With these explanations, each convolution block in the proposed CNN models can be configured using three optimization variables: the size of the convolution filter, the number of convolution filters, and the type of pooling function. Since the proposed CNN model includes 5 convolution blocks; Therefore, the number of optimization variables in the problem discussed in this research will be equal to 15. In other words, each solution vector in BHO algorithm is described as a numerical vector with length of 15. In each solution vector, the first 5 variables are determined as a natural number in the interval [4,16], which represent the determined values for the size (length and width) of each convolution filter (values $\left\{w_{1},\ldots ,w_{5}\right\}$ and $\left\{L_{1},\ldots ,L_{5}\right\}$ in Fig. 2). Also, the second 5 variables of each solution vector represent the number of filters considered for each convolution layer (values $\left\{N_{1},\ldots ,N_{5}\right\}$ in Fig. 2) which include natural numbers in the interval [4,64]. Finally, the last five variables that accept one of the numbers 1 or 2, are used to describe the type of pooling function considered for each convolution block.

BHO algorithm has been used to optimize the configuration of each CNN model. The fitness of each solution vector has been evaluated using the training error criteria. To achieve this objective, the first step involves applying a solution vector, denoted as x, to the configuration of the fundamental CNN model. Subsequently, the hyperparameters of the model are fine-tuned using the values obtained in x. Subsequently, a SoftMax layer is appended to the final stage of this Convolutional Neural Network (CNN) model in order to compute the training error of the CNN model. Finally, the fitness of the solution *x* is calculated based on the training samples as follows:

(5) $$fitness\left(x\right)=\frac{F}{Tr}$$

where F is the number of training samples for which the CNN model's assigned label differs from the sample's true label. Additionally, Tr shows how many training samples there are overall. Given that the CNN model that is constructed based on each candidate solution's fitness must be trained over an extended period of time, Therefore, in this step, a small set of training samples has been used. This research, uses the BHO algorithm proposed by Hatamlou [26] for optimizing the hyper-parameters of the CCN models. The BHO algorithm is an optimization strategy for solving problems by modeling the motion of stars and black holes. In BHO, each candidate solution is considered as a star, which is determined based on its position in the *n*-dimensional space (*n* determines the dimensions of the problem). The algorithm starts by determining a random position for *p* stars in the problem space. The group of stars that were created from the original population. Following formation, the fitness function (equation (5)) is used to determine each star's quality; the star with the highest fitness is said to be a black hole. BHO uses an iteration-based procedure to explore the problem space and mimic stellar motion. Thus, the position of each star such as *X*_*i*_ is updated using equation (6) [26]:

(6) $$X_{i}=X_{i}+rand.\left(X_{BH}-X_{i}\right)$$

where, $X_{BH}$ indicates the position of the black hole and $X_{i}$ indicates the position of the star i. Eq. (6) guarantees effective search in the problem space but still requires a strategy to avoid getting trapped in local optima. To solve this problem, in BHO a threshold such as *R* is used as the threshold distance for being swallowed by a black hole [26]:

(7) $$R=\frac{f_{BH}}{\sum_{i=1}^{N}f_{i}}$$

in equation (7), $f_{BH}$ and $f_{i}$ represent the fitness of the black hole and the fitness of the solution i, respectively. Also, *N* specifies the size of the population in the optimization algorithm. Thus, if the distance of a star like *X* to the black hole is less than the threshold *R*, then *X* is replaced by a new random star in the problem space. This process will be repeated until one of the termination conditions of the algorithm is met. Considering the described structure for each solution vector and fitness function, the optimization steps of each CNN model in the proposed method using the BHO algorithm can be summarized as follows:

I. Determine the initial population of the optimization algorithm, randomly.
II. Evaluate the fitness of each solution using Eq. (5).
III. Find the star (solution) with the minimum fitness and store it as a black hole $\left(X_{BH}\right)$.
IV. Update the new location for each star $X_{i}$ using Eq. (6).
V. Calculate the value of the threshold *R* to estimate the distance of a star being swallowed by the black hole, using Eq. (7).
VI. If a star such as $X_{i}$ has a lower fitness than $X_{BH}$; Then $X_{BH}=X_{i}$ and $f_{BH}=f_{i}$.
VII. If the distance of a star such as ${\;X}_{i}$ from the black hole is less than *R*; then replace $X_{i}$ with a new random solution.
VIII. If the fitness of the best solution reaches zero or the number of iterations of the algorithm has reached the threshold *T*, then terminate the algorithm; otherwise, repeat the algorithm from step (II).

By implementing the above process, a suitable configuration will be created for each CNN model, and these configured models will be used to process test samples and recognize music genres.

### 3.4 Classification and recognition of musical styles

The last stage of the suggested approach involves concatenating the features that were retrieved by each CNN model to create the final features that describe the properties of the signal. For this, the generated models are utilized to extract the features of the test samples after the BHO algorithm optimizes the hyperparameters of two CNN models based on the training data. In this case, each test sample (similar to the training phase) is pre-processed and then its MFCC and STFT matrices are extracted. Each of these matrices are given to the optimized CNN model so that the features of the test sample are obtained based on the output values of the last fully connected layer of each CNN model. Each CNN model in the proposed architecture is finalized by a fully connected layer including 200 neurons which represent the features extracted from input. Therefore, each CNN model produces an output of dimension 200 × 1. These outputs are merged through a concatenation layer in the form of a vector to construct a vector with dimension of 400 × 1, and finally a SoftMax-based classifier is used to classify these features. In order to do this, a fully connected layer of C neurons receives the concatenated features. C neurons decide how many music genre classes there are. Based on these characteristics, a SoftMax layer is trained to provide a probability distribution across the potential classes. The test sample will be a member of the class whose associated output neuron has the greatest probability value in this classification model, where the number of output neurons is equal to C (music genres).

## 4 Simulation and results

MATLAB 2018a software has been used to accomplish the suggested approach. Two databases were employed for the experiments: Extended-Ballroom [27] and GTZAN [28]. Additionally, the accuracy, precision, recall, and F-Measure criteria were used to assess the efficacy of the proposed technique in various scenarios. The resulting data were then compared with those from earlier studies on the categorization of musical genres. The features of the evaluation measures and databases that were employed will be described first, followed by a discussion of the outcomes.

### 4.1 Database and evaluation metrics

Two databases GTZAN [28] and Extended-Ballroom [27] have been used in the implementation of the proposed method. The GTZAN database is one of the first and most widely used databases for the classification of music genres. There are a thousand 30-s music recordings in this collection that were recorded at a frequency of 21.5 kHz. All of GTZAN's songs are 16-bit mono audio files kept in.wav format. The 10 music genres included in the GTZAN database are pop, reggae, rock, hip-hop, jazz, blues, pop, country, disco, and pop. Additionally, there are 100 examples in this database for each category. A more recent and comprehensive dataset for the categorization of musical genres is the Extended-Ballroom database. With 4180 entries, this database is an altered version of the Ballroom dataset. Every sample in this database has a duration of 30 s, and it falls into one of the 13 categories that are accessible. The target classes in this database are: cha-cha (455), jive (350), quickstep (497), rumba (470), samba (468), tango (464), Viennese waltz (252), waltz (529), foxtrot (507), pasodoble (53), salsa (47), slow waltz (65) and wcswing (23).

Every sample from the two aforementioned databases was utilized in the studies. The 10-fold cross validation methodology, in which the samples are split into 10 sections and the tests are performed 10 times, has been used to increase the validity of the findings and guarantee the correctness of the suggested procedure under various settings. Every iteration employs a fresh segment to evaluate the categorization model. Each test sample's projected label for the musical genre is compared to the sample's actual label. Based on the comparisons, the accuracy, precision, recall, and F-Measure criteria are used to measure the classification model's efficiency. The accuracy criterion, which is determined by dividing the number of properly categorized test samples by the total number of test samples, displays the percentage of correctly identified test samples. In multi-class situations, the precision criterion characterizes the accuracy of the detection method for each class and shows the number of successfully categorized samples in each target class. This criterion is calculated using equation (8).

(8) $$Precision=\frac{TP}{TP+FP}$$

The number of occurrences of the examined class whose label is accurately predicted is represented by TP in the equation above. However, FP indicates the quantity of samples that are really part of another class but were mistakenly assigned to the class that is being studied. The recall criteria is used to characterize the percentage of positive class samples that have accurate labels. $Recall=\frac{TP}{TP+FN}$ (9)

In equation (9), FN refers to the number of samples belonging to the studied class which were incorrectly classified in other classes. Finally, the F-Measure can be calculated as the harmonic mean of precision and recall criteria, using equation (10).

(10) $$F-Measure=2\times \frac{Precision\times Recall}{Precision+Recall}$$

The accuracy, recall, and F-Measure criteria are computed individually for every class throughout the trials, and the average values of these criteria are also looked at for all of the target classes. The BHO algorithm is used in the suggested strategy to optimize the CNN model's hyperparameters, as previously mentioned. The population size (the total number of stars in BHO) and the number of optimization method iterations are set to 50 and 250, respectively, in both scenarios. All the tests were done on a personal computer, running Microsoft Windows 10 on an Intel Core i7 processor with a frequency of 3.2 GHz and 32 GB of main memory. This processor has 6 physical cores, whose parallel processing capability is used for the simultaneous optimization of two CNN models by the BHO algorithm. On the other hand, based on the processing capabilities of MATLAB software, training of CNN models has been done based on CUDA technology and on an Nvidia GeForce GTX 1080 graphics card.

### 4.2 Results and discussion

This part continues with a discussion of the findings from the assessment of the suggested approach using the scenario from the preceding section. To get a thorough assessment of the suggested approach's performance, its effectiveness has been examined across various functional conditions. These situations include:

1. Performance of the proposed method based on MFCC and STFT features
2. Performance of the proposed method based on MFCC features
3. Performance of the proposed method based on STFT features
4. Performance of the proposed method without optimization of CNN models by BHO

In the first scenario, the classification of music genres is done based on the combination of MFCC and STFT features and using two CNN models corresponding to them. In the second scenario, only MFCC features and a CNN model were used to classify music genres. The third scenario in the above list refers to the condition that the evaluation is done only based on STFT features and a CNN model. The fourth scenario, which is the last one, specifies that the BHO algorithm will only be used to assess the efficacy of the deep learning models' optimization strategy in the suggested manner, rather than to optimize the hyperparameters of the two proposed CNN models.

It should be mentioned that, in addition to the scenarios mentioned above, the effectiveness of the suggested approach has also been contrasted with the BAG technique offered by Prabhakar et al. in Ref. [19] and the way of Yang et al. in Ref. [15]. These two recent studies have been selected to be compared with the suggested technique in the experiments since they have reported better performance than the other studies. Table 1 shows an example of the best configuration that the BHO algorithm found for each of the CNN models that are used in the suggested approach. The data shown in Table 1 indicates that although the two CNN models used in the suggested approach are designed according to similar principles, there are variations in the specifics of their setup.

**Table 1:** An example of the optimal configuration discovered by the BHO for the proposed CNNs.

Layer	CNN_MFCC_	CNN_STFT_
Convolution_1_ (W × L,N)	16 × 16,20	15 × 15,31
Pooling_1_	Max Pooling	Max Pooling
Convolution_2_ (W × L,N)	13 × 13,22	11 × 11,38
Pooling_2_	Max Pooling	Max Pooling
Convolution_3_ (W × L,N)	8 × 8,26	9 × 9,43
Pooling_3_	Max Pooling	Max Pooling
Convolution_4_ (W × L,N)	7 × 7,38	6 × 6,57
Pooling_4_	Max Pooling	Average Pooling
Convolution_5_ (W × L,N)	4 × 4,39	3 × 3,64
Pooling_5_	Average Pooling	Average Pooling

The number of filters rises and their dimensions’ decrease with the advancement of the convolution layers in both CNN models. Because in the deeper layers of each CNN model, the complexity of the patterns in the feature maps increases, and in order to more fully extract the patterns related to music genres from MFCC/STFT data, it is necessary to increase the diversity of feature map combinations. On the other hand, in both CNN models, the final pooling layers are determined as average and the initial layers are of max pooling type. On the other hand, each convolution layer in the CNN model used for processing STFT of signals, generally includes more convolution filters with smaller dimensions compared to the other CNN. This feature can indicate the higher complexity of the patterns in the STFT matrix of signal compared to its MFCC matrix.

**Fig. 3:**
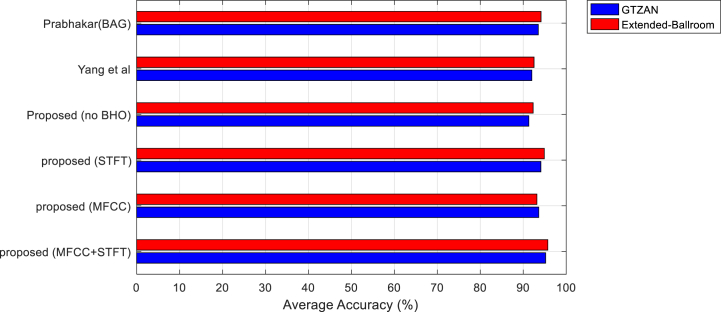
The average accuracy of the proposed method and other methods in the classification of music genres for GTZAN and Extended-Ballroom databases.

Fig. 3 compares the average accuracy of the suggested strategy with other approaches for the GTZAN and Extended-Ballroom databases' music genre categorization. The Extended-Ballroom database has a generally more accurate categorization of musical genres than the GTZAN database. Since one of the fundamental needs of deep learning models is that the Extended-Ballroom database has almost four times as many target samples than the GTZAN database, even though it has more target classes. That is, it meets the need for a high number of training samples. Based on this figure, the suggested technique has an average accuracy of 95.2 % in classifying GTZAN database samples. On the other hand, the suggested technique's accuracy for the Extended-Ballroom database is 95.72 %, and in both of these cases, the proposed approach is better in terms of accuracy. When the accuracy criteria are examined, it is discovered that employing STFT features (while disregarding the CNN model based on MFCC) might result in greater accuracy than using MFCC features. Despite the fact that each of these circumstances has a greater average accuracy than the two approaches of Yang et al. [15] and BAG [19]. The suggested method's increased accuracy may be ascribed to the BHO algorithm's usage of the CNN model optimization technique. Because if this strategy is not used in the proposed method ("no BHO" mode), the classification accuracy for GTZAN and Extended-Ballroom databases is 91.3 and 92.29, respectively, implying that the CNN models optimization strategy in the proposed method increases the accuracy by at least 3.4 %.

**Fig. 4:**
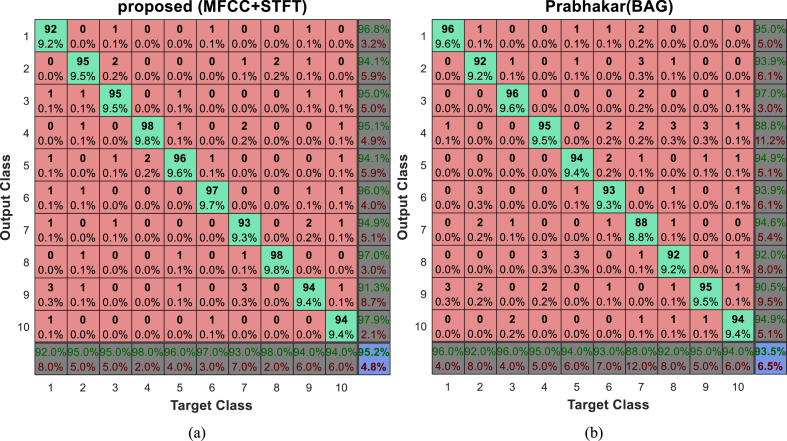
Confusion matrix of (a) proposed method and (b) BAG method in classification of GTZAN database samples.

The BAG technique [19], which uses a mixture of BiLSTM and GCN to classify music genres, performs the closest to the suggested method, as shown in Fig. 3. More specific information regarding the ways in which various genres may be identified using classification techniques can be found in the confusion matrix. The suggested method's confusion matrix and the BAG method's for classifying GTZAN database samples are shown in Fig. 4.

**Fig. 5:**
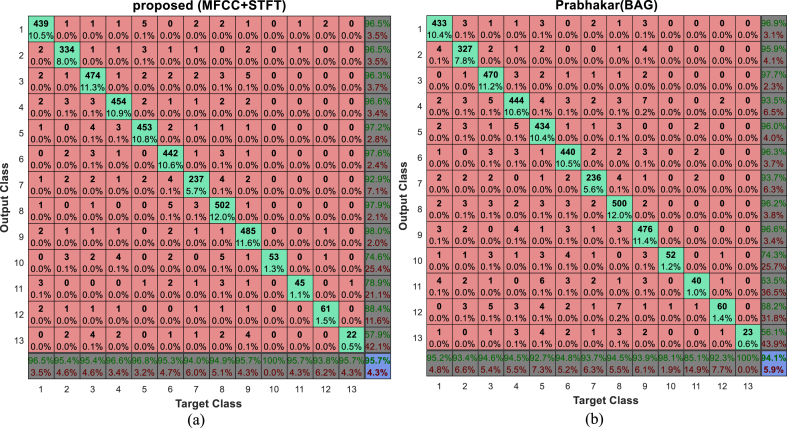
Confusion matrix of (a) proposed method and (b) BAG method in classification of Extended-Ballroom database samples.

Numbers 1 through 10 stand for, successively, blues, classical, country, disco, hip-hop, jazz, metal, pop, reggae, and rock labels in Fig. 4's confusion matrices. The test sample labels are shown in each column of the matrix, while the sample labels according to each classification technique are displayed in the matrix's rows. As an example, Fig. 4-a shows that, using the suggested technique, 92 samples out of 100 samples that belong to the Blues class (sum of the first column of the matrix) are properly categorized. Also, three examples are wrongly classified in the reggae category. On the other hand, the proposed method has placed 95 samples in the blues category (the sum of the values of the first row of the matrix), among which 3 samples belonged to country, jazz and reggae classes. Interpretation of the results for other categories in this matrix is done in a similar way. Comparison of Fig. 4-a and 4-b shows that the BAG method works better than the proposed method only in the classification of blues, country and reggae samples and the proposed method of other music genres classifies more accurately than the BAG method, after which the accuracy will increase by 1.7%. Fig. 5-a and 5-b show the confusion matrices of the proposed method and the BAG method in classification of Extended-Ballroom database samples, respectively.

Additionally, in Fig. 5, the designations cha-cha, jive, quickstep, rumba, samba, tango, Viennese waltz, foxtrot, pasodoble, salsa, slow waltz, and wcswing are correspondingly represented by the numbers 1 through 13 in the confusion matrix. In this instance, the proposed method reports a better performance than the BAG method in the classification of the first 12 classes, and based on that, it can increase the classification accuracy by at least 1.6% compared to this method. This is because the BAG method is the method that is closest to the proposed solution when it comes to classifying different genres for the Extended-Ballroom database. We may evaluate how well various approaches categorize distinct genres of music using confusion matrices, taking into account recall and accuracy standards for the Extended-Ballroom and GTZAN databases. It is evident from looking at the confusion matrices in Fig. 4, Fig. 5 that the suggested approach, when using MFCC and STFT characteristics, is more effective than other approaches at classifying distinct genres. The suggested technique only outperforms the examined methods in terms of overall quality for the blues genre categorization in the GTZAN database. However, only the wcswing class in the Extended-Ballroom database shows a drop in classification quality when compared to other approaches; in all other classes, the suggested method performs more efficiently when it comes to identifying musical genres.

**Fig. 6:**
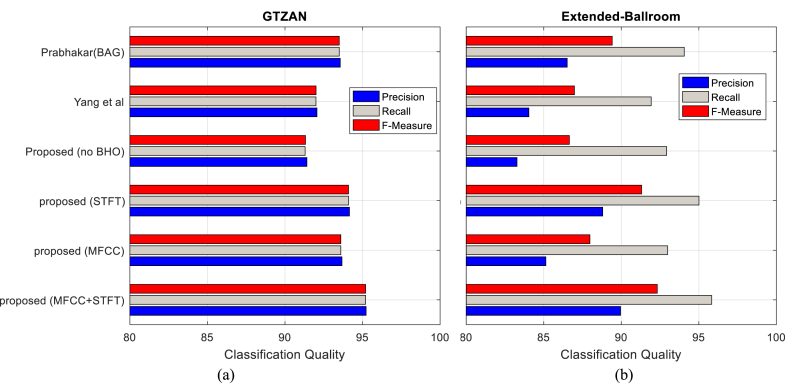
The average values of precision, recall and F-Measure criteria for (a) GTZAN and (b) Extended-Ballroom databases.

In Fig. 6, the average precision, recall and F-Measure measures are calculated. Fig. 6-a reports these criteria obtained through GTZAN database, while Fig. 6-b reports these results obtained through Extended-Ballroom database. These diagrams show the general performance of different methods in terms of classification quality.

**Fig. 7:**
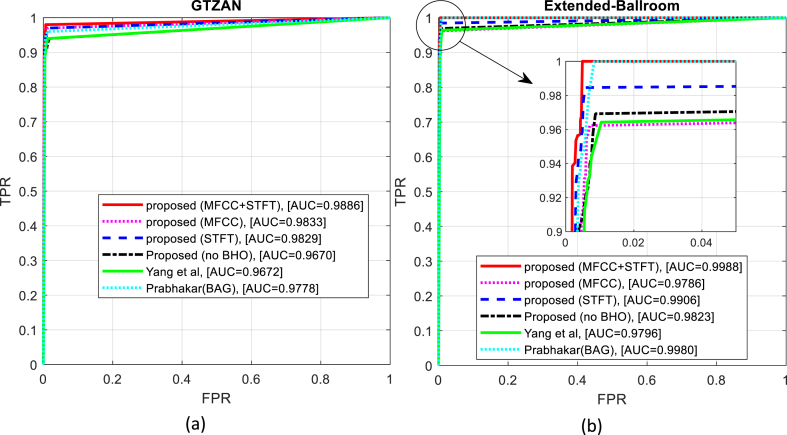
ROC curve resulting from the classification of (a) GTZAN and (b) Extended-Ballroom database samples.

The Received Operating Characteristics (ROC) curves obtained from the genre categorization of the two tested datasets, GTZAN and Extended-Ballroom, are shown in Fig. 7-a and 7-b, respectively. This diagram shows that the suggested technique outperforms the comparable methods in terms of area under the curve (AUC), true positive rate (TPR) and false positive rate (FPR). Therefore, it can be said that the approach suggested in this study has a greater average accuracy in terms of accurately classifying musical genres.

Table 2 provides the numerical outcomes of the tests carried out in this section. The suggested approach may identify music genres with a greater quality than the tested approaches, as shown by the comparison of accuracy, precision, recall, and AUC criteria in Table 2. These findings suggest that the suggested strategy may enhance accuracy, recall, and F-Measure criteria in the GTZAN and Extended-Ballroom databases. With a better chance of accuracy than other approaches, the suggested method's higher precision validates that the outputs generated for each genre are accurate. However, greater recall demonstrates that a larger percentage of samples accurately identified as belonging to musical genres was possible using the suggested approach.

**Table 2:** Comparing the efficiency of the proposed method with other methods.

Database	Method	Accuracy	F-measure	Recall	precision	AUC
**GTZAN**	**Proposed (MFCC** + **STFT)**	95.2000	95.1986	95.2000	95.2311	0.9886
	**Proposed (MFCC only)**	93.6000	93.6030	93.6000	93.6739	0.9833
	**Proposed (STFT only)**	94.1000	94.0999	94.1000	94.1572	0.9829
	**Proposed (no BHO)**	91.3000	91.3230	91.3000	91.4119	0.9670
	**Yang et al** [15]	92	92.0042	92	92.0605	0.9672
	**Prabhakar et al (BAG)** [19]	93.5000	93.5008	93.5000	93.5620	0.9778
**Extended Ballroom**	**Proposed (MFCC** + **STFT)**	95.7177	92.3202	95.8293	89.9544	0.9988
	**Proposed (MFCC only)**	93.1818	87.9783	92.9890	85.1381	0.9786
	**Proposed (STFT only)**	94.9043	91.3120	95.0112	88.8033	0.9906
	**Proposed (no BHO)**	92.2967	86.6498	92.9301	83.2777	0.9823
	**Yang et al** [15]	92.5120	86.9798	91.9294	84.0380	0.9796
	**Prabhakar et al (BAG)** [19]	94.1388	89.4153	94.0595	86.5123	0.9980

## 5 Conclusion

The classification of music genres is one of the basic components in applications such as music information retrieval. Classification of music songs, artists, albums, etc. based on their common genre characteristics can be a useful solution for organizing large databases. For this reason, the issue of classification of music genres is of considerable importance. In this article, a new method for classifying music genres using deep learning techniques was presented. The proposed strategy provides a parallel framework of CNN models in which each CNN model is used to process a specific set of signal features. As shown, a more comprehensive collection of signal properties may be obtained by the successful use of the suggested hybrid method. Conversely, the BHO algorithm is used in the suggested approach to optimize each CNN model's hyperparameters. CNN models are able to dynamically modify their configuration based on the characteristics that are fed into them thanks to this approach. According to study results, using the BHO method to optimize CNN model design may raise detection accuracy by at least 3.4 %. Using the GTZAN and Extended-Ballroom databases, the usefulness of the suggested approach for categorizing musical genres was examined. Based on the findings, the suggested strategy may be more effective than the earlier approaches in accurately identifying each genre independently, in addition to improving overall detection accuracy. These findings demonstrate that the suggested technique can identify all genres of music in the Extended-Ballroom and GTZAN databases with accuracies of 95.7 % and 95.2 %, respectively. This is an improvement in accuracy of 1.7 % and 3.6 % over the nearest method. The suggested method's need for additional time to train the learning models is one of its drawbacks. Even if each CNN model's ideal configuration uses the parallel processing technique in the suggested manner, this time difference may be significantly decreased by constructing a parallelized version of the BHO algorithm. The proposed model showed an acceptable performance in recognizing the main genres of music. However; Each genre of music includes subgenres that the proposed model should be able to distinguish in order to be used in real-world situations. Therefore, the development of the proposed method for the purpose of recognizing music subgenres is one of the issues that can be addressed in future works. The proposed solution can be extended to similar problems such as: speech language recognition or emotion recognition in speech; Future study might thus examine how well the suggested approach performs in resolving these issues. The research's approach may be used to automatically classify music genres in large datasets. Additionally, by enlarging the search boundaries of the optimization method used in this study, the problem space may be expanded in an effort to produce a model for music genre categorization that is more accurate.

## Data availability statement

The data that support the findings of this study are openly available in the following repositories:

• GTZAN: https://www.kaggle.com/datasets/andradaolteanu/gtzan-dataset-music-genre-classification
• Extended-Ballroom: http://anasynth.ircam.fr/home/media/ExtendedBallroom

The datasets are publicly accessible without restrictions.

## CRediT authorship contribution statement

**Teng Li:** Resources, Methodology, Investigation.

## Declaration of competing interest

The authors declare that they have no known competing financial interests or personal relationships that could have appeared to influence the work reported in this paper.
